# Thermal petiole wounding triggers trap closure in *Dionaea muscipula*

**DOI:** 10.1080/15592324.2026.2700900

**Published:** 2026-07-09

**Authors:** Neleh Bergstrom, Paul Helfrich

**Affiliations:** a Department of Biology, University of Montana Western, Dillon, MT, USA; b Department of Biology, University of Mary, Bismarck, ND, USA

**Keywords:** *Dionaea muscipula*, action potential, signaling, bioelectricity, electrophysiology

## Abstract

The Venus flytrap (*Dionaea muscipula* Ellis) is a carnivorous plant that captures invertebrates using an electrically excitable trap mechanism. Invertebrates stimulate trigger hairs, initiating action potentials (APs) that drive rapid closure. Trap lobes are electrically isolated from one another by non-excitable petioles. We examined thermal wounding to leaf petiole's ability to produce detectable electrical activity in the isolated trap and if wounding stimulation is sufficient to trigger closure. Using a within-subject design across 15 mature plants, we compared trap-recorded electrical signals following trigger hair (TH) stimulation and petiole branding with a heated soldering iron. Primary spike morphology did not differ significantly between treatment groups for either Spike/Baseline Ratio or Depolarization Fraction. Within-Brands primary-to-secondary Spike/Baseline Ratios differed significantly consistent with non-regenerative signal attenuation rather than canonical AP propagation from the petiole. Differences are consistent with thermal petiole wounding disrupting xylem continuity and generating a hydraulic pressure transient that initiates trap-derived APs through the trap's own excitation machinery. The trap's closure threshold is accessible via sufficiently large, likely non-electrical, systemic perturbation.

## Introduction

All organisms must precisely coordinate external environmental stimuli with adaptive physiological responses.^1^ Eukaryotic plasma membranes function as dynamic interfaces for signal transduction.^2^ Electrochemical gradients produced by asymmetric ion distribution across the cell periphery generate substantial electric potential.^3^ Electrical signals propagating through tissues initiate physiological outcomes.^4^ Though often overlooked in non-animal systems, electrically driven cellular communication is deeply embedded in the evolutionary history of plants.^5^ Scientific interest in plant bioelectricity dates to at least the 1700s,^6–8^ and current data suggest extensive bioelectrical capacity across the clade.^3^ ^,^ ^5^ ^,^ ^9^ Indeed, recent electrophysiological surveys documented detectable electrical responses to touch and flame stimuli in approximately 60% of species tested.^10^

The Venus Flytrap (VFT), *Dionaea muscipula* Ellis, is the best-characterized bioelectric model plant.^9^ Action potentials (APs) propagate through leaf-derived traps in response to specialized trigger hair deflection by captured invertebrates.^11^ ^,^ ^12^ VFTs scale their physiological responses to AP generation through rapid trap closure, jasmonate (JA) signaling, and digestive enzyme secretion to best extract nutrients from captured prey.^13–15^

Central to coordinated responses in VFTs is individual trap electrical isolation^6^ ^,16^ . Electrical isolation minimizes metabolic costs associated with invertebrate digestion as stimulated traps reduce local photosynthesis and stimulate respiration.^17^ Trap petioles act as bioelectric buffers as they are non-excitable and do not propagate APs.^18^ Trap excision from the petiole does not impair AP generation or closure.^9^ Prey capture in one trap does not produce JA accumulation or digestive responses in adjacent traps on the same plant, further supporting trap-level isolation and functional autonomy.^19^

Although individual traps maintain electrical isolation under normal conditions, there is evidence in other plants that thermal injury can drive systemic variation potential (VP) propagation in injured tissues.^5^ ^,^ ^20^ VPs are graded, non-regenerative signals thought to result from transient H + -ATPase inactivation and hydraulic pressure wave propagation through xylem.^5^ ^,^ ^21^ Recent experiments using laboratory simulated brushfires and lit matches indicate that heat challenged plants close their traps during stress but retain the ability to capture prey.^22^ Burning-induced VPs can produce measurable physiological effects in distal tissues.^21^ Whether VFT petiole thermal wounding generates similar electrical perturbations sufficient to activate the electrically isolated trap is unexamined. Here we report exploratory within-subject results in which thermal petiole wounding was compared with canonical trigger hair stimulation in terms via both trap-recorded electrical signal morphology and closure outcomes in VFTs.

## Materials and methods

### Plant material and experimental design

Mature *Dionaea muscipula* Ellis specimens (*n =* 15) were acclimated to controlled laboratory conditions for 6 weeks inside a closed 38-L (10-gallon) glass terrarium prior to experimentation. Plants were cultivated in 7.6-cm (3-inch) pots using a commercial carnivorous plant substrate (75% sphagnum moss, 25% perlite; Gardenera, USA). Substrate saturation was maintained by partial (~20%) pot base submersion in deionized water (~18 MΩ·cm resistivity). Photoperiod was regulated at 16:8 h light:dark via a full-spectrum LED grow light (LBW Desk Grow Light), and constant ambient humidity and root-zone warmth were provided by a beneath-tank substrate heating mat (BN-LINK).

Only mature traps displaying prominent red pigmentation on the adaxial lobe surfaces and an aggregate lobe length >4 cm were selected for experimental trials. To minimize the inter-specimen phenotypic variability, a within-subject experimental design was implemented wherein every individual plant received both experimental treatments (mechanical trigger hair stimulation and thermal petiole wounding). Individual traps were subjected to a single experimental trial to avoid mechanical or physiological fatigue.

### Electrophysiological recordings

Extracellular bioelectric activity was recorded using a dual-channel Plant SpikerBox amplifier (Backyard Brains, Ann Arbor, MI) through the ‘SpikeRecorder’ software set to ‘plant’ according to manufacturer instructions. A single stainless steel recording electrode was fixed to the outer target trap lobe, using the standard configurations and coated with conductive SignaGel (Parker Laboratories).^10^ All measurements were grounded using the associated pin wire applied to the pot substrate. Signal acquisition was captured as uncompressed 16-bit mono PCM waveforms (.wav) at a 44,100 Hz sampling rate. Raw signal amplitude is expressed in arbitrary units (A.U.) linearly proportional to transmembrane voltage (Backyard Brains, personal communication). All amplitude-dependent comparative metrics are expressed as dimensionless internal ratios, canceling out the instrument's uncalibrated absolute voltage constant and validating multi-plant statistical integration.^10^

Experimental sessions were conducted in a controlled environment with all non-essential alternating current (AC) electrical appliances disconnected to isolate the recording field from ambient electromagnetic interference. Continuous tracking was initiated prior to physical manipulation to establish a stable, steady-state baseline. Baseline root-mean-square (RMS) amplitude was mathematically defined from a 5-second pre-stimulus window.

### Trigger hair & thermal petiole wounding

Specimens were moved from the humidity terrarium to a bench immediately prior to data collection. Two consecutive manual single adaxial trigger hair deflections were delivered via a blunt-tipped plastic stylus for each mechanical trigger hair (TH) stimulation. Deflections were spaced at an approximate 3-second inter-stimulus interval to allow localized membrane potential recovery. Trials characterized by structural electrode displacement or tissue contact loss during trap closure were discarded from waveform analysis (*n =* 4), resulting in *n =* 11 validated TH electrical recordings for subsequent morphological analysis.

Thermal petiole wounding (“branding”) was executed using a heavy-duty dual-heat soldering gun (Weller Professional, 200 W rated capacity). The tool tip was brought to maximum thermal equilibrium under a standardized 30-second free-air heating window to achieve a maximum 593 °C (1100 °F) uniform tip temperature. Immediately prior to tissue contact, the iron was disconnected from the main AC power supply to eliminate electrical line noise contamination within the active recording channel. The heated element was applied directly to the petiole blade center immediately below the targeted trap lobe structure. Contact was sustained for 8 seconds or until all visible movement ceased.

To isolate direct tissue branding effects from the convective or radiant heat, an independent control series was performed (*n =* 10). The fully heated element (593 °C) was held <1 cm from trap lobes and petiole margins for 8 seconds without establishing physical contact. No detectable bioelectric signal artifacts or macroscopic trap closure responses were observed across any control replicates, confirming that the responses observed during direct branding require physical tissue disruption.

### Signal processing and waveform analysis

Raw bioelectric data were processed via a custom pipeline implemented in Python. Waveforms were passed through a 4th-order digital Butterworth bandpass filter (0.5–50 Hz) to eliminate low-frequency DC drift and high-frequency thermal noise while preserving the characteristic ~1-second depolarization *Dionaea* action potential.^23^ Individual spike events were programmatically isolated using a peak-detection threshold exceeding 2.5 standard deviations above the bandpass-filtered signal mean, with a 0.3-second minimum refractory distance enforced to prevent transient multi-peak counting.

For each identified spike, a 1-second analysis window centered on the peak was extracted. Events were binned chronologically as Primary (index = 1) or Secondary (index > = 2). The dimensionless Spike/Baseline Ratio was computed by dividing the 1-second spike window RMS amplitude by the pre-stimulus RMS amplitude baseline window. Depolarization Fraction was calculated as |peak|/Peak-to-Peak (P2P) voltage within the filtered spike window. Mean waveforms were constructed and plotted by extracting a window around each detected spike, normalizing each trace to its own peak-to-peak amplitude, and averaging across spikes within each treatment × spike order category. The display window was framed from −0.2 to 0.6 s relative to peak depolarization to capture the full asymmetric AP event from onset through repolarization recovery. High-leverage outlier points (*n =* 2) corresponding to incomplete waveform acquisition were excluded from the final computational dataset.

### Kinematic closure analysis

Trap closure kinetics were quantified using digital video analysis. High-resolution video recordings were captured perpendicular to the trap movement plane, starting >3 s pre-stimulus and continuing until >3 s post-stabilization. Individual frames were extracted at fixed 0.1-second intervals. The spatial pixel distance between corresponding coordinate markers on the opposing trap lobes margin apices was tracked using digital coordinate tracing, consistent with established kinematic models.^24^ Kinematic closure progress was normalized using lobe separation to initial baseline opening ratios.

The primary kinetic metric evaluated was the elapsed time required to achieve 50% closure (T50). To classify distinct closing profiles observed during the wounding trials, a 1.5 second empirical threshold, corresponding to a natural gap in the aggregate T50 distribution, was used to post-hoc categorize trials into Brands-Fast (T50 < 1.5 s) or Brands-Delayed (T50 > 1.5 s) sub-types.

### Statistical analysis

Statistical evaluation was conducted using Welch’s t-tests to accommodate heteroscedasticity across the experimental cohorts, with effect magnitudes calculated via Cohen’s d. Three primary comparisons were designated *a priori* to test signal equivalence and attenuation: (1) Primary Spike/Baseline Ratio between TH and Brands cohorts; (2) Primary Depolarization Fraction between TH and Brands cohorts; and (3) Intra-treatment Primary versus pooled Secondary Spike/Baseline Ratios within the Brands cohort. All pre-specified primary endpoints were evaluated at alpha = 0.05. All supplementary pairwise metrics were designated exploratory, with nominal *p*-values reported alongside conservative Holm–Bonferroni adjusted family-wise alpha thresholds.

## Results

### Petiole branding produces trap closure

**Figure 1.**
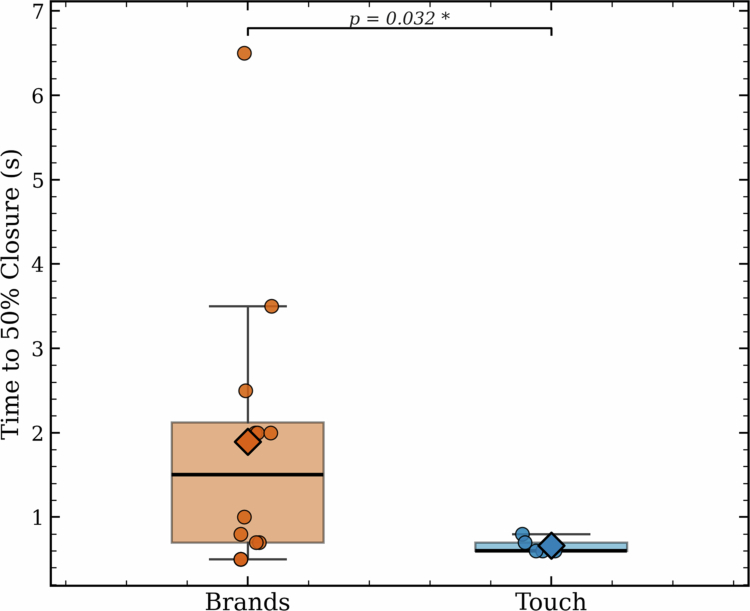
Speed of trap closure arranged by treatment. Boxes show data tendency (median, IQR, whiskers to 10th/90th percentile) with individual data points overlaid showing time to 50% closure (s) for Brands and Touch (trigger hair) stimulation (closing trials only; Brands *n =* 12, Touch *n =* 5). Diamond symbols indicate group means. Brands closure was significantly slower than Touch driven closure (Welch's t, *p =* 0.032, d = 1.01).

Petiole thermal branding produced trap closure in 12/19 trials (63%), while seven trials did not achieve closure, maintaining a pixel ratio > = 0.85 throughout the recording window (Supplementary Data Sheet 4). Traps achieving closure reached substantial closure, showing a > = 80% reduction in baseline opening in both treatment groups (Supplementary Data Sheet 4). Among closing trials, time to 50% closure was significantly longer in the Brands group than in Touch stimulation (Brands: *n =* 12, mean = 1.9 s, SD = 1.7 s; Touch: *n =* 5, mean = 0.7 s, SD = 0.09 s; Welch's t, *p =* 0.032, d = 1.01; Figure 1).

**Figure 2.**
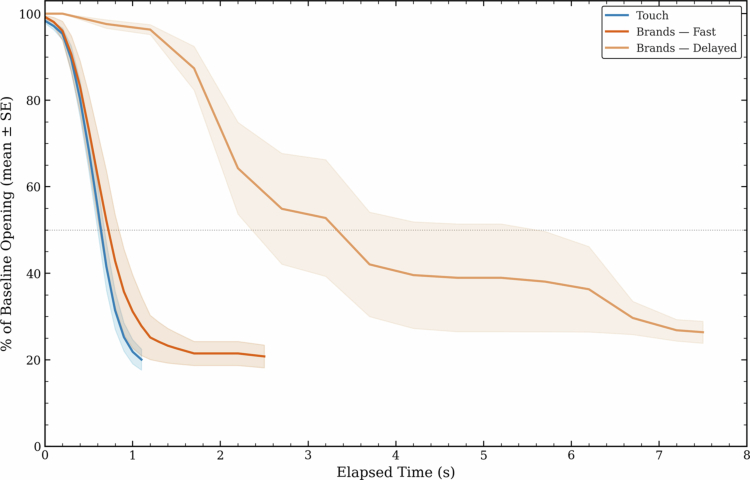
Mean closure dynamics arranged by response group. Lines represent mean + /- SE percentage of baseline opening over elapsed time (s) for three groups: Touch (*n =* 5, blue), Brands-Fast (*n =* 6, dark orange), and Brands-Delayed (*n =* 6, light orange). Groups were defined post-hoc at the natural gap in the T50 distribution (<= 1.5 s = Fast; >1.5 s = Delayed). Only trials achieving closure (pixel ratio <= 0.50 at any measured timepoint) are included. The dotted horizontal line indicates 50% of baseline opening.

Individual closure trajectories revealed heterogeneity within the Brands group. A trial subset produced rapid closure within approximately 1.5 seconds (Brands-Fast; *n =* 6; Figure 2). The remaining closing trials exhibited a delayed closure pattern, with traps closing gradually over 2 to 7 seconds (Brands-Delayed; *n =* 6; Figure 2).

**Figure 3.**
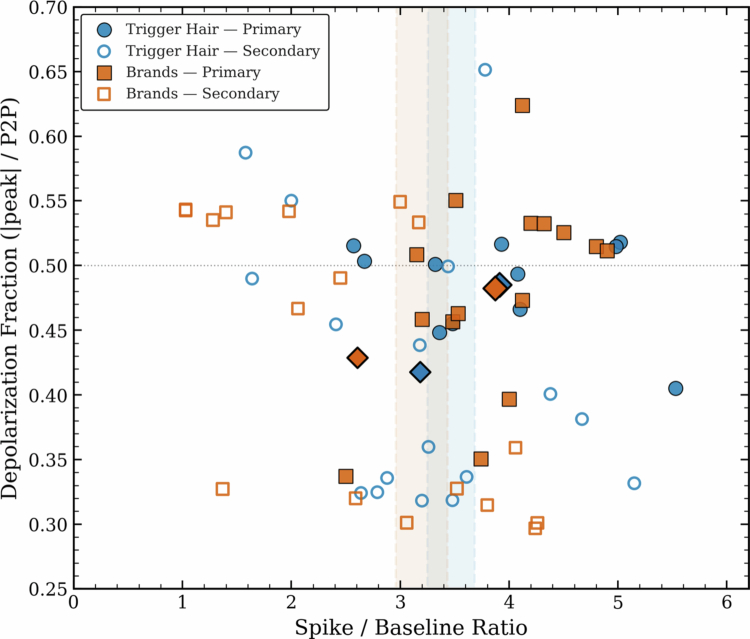
Trigger Hair vs. Brands spike profiles arranged as Depolarization Fraction (|peak|/P2P) vs. Spike/Baseline Ratio for all individual spikes. Filled symbols = Primary spikes; open symbols = Secondary spikes. Blue circles = Trigger Hair; orange squares = Brands. Large black diamonds indicate group means for Primary spikes (each treatment). Shaded vertical bands indicate the mean + /- SD range for Brands Primary (orange) and Trigger Hair Primary (blue) Spike/Baseline Ratios.

### Spike morphology differences between treatments and order

**Figure 4.**
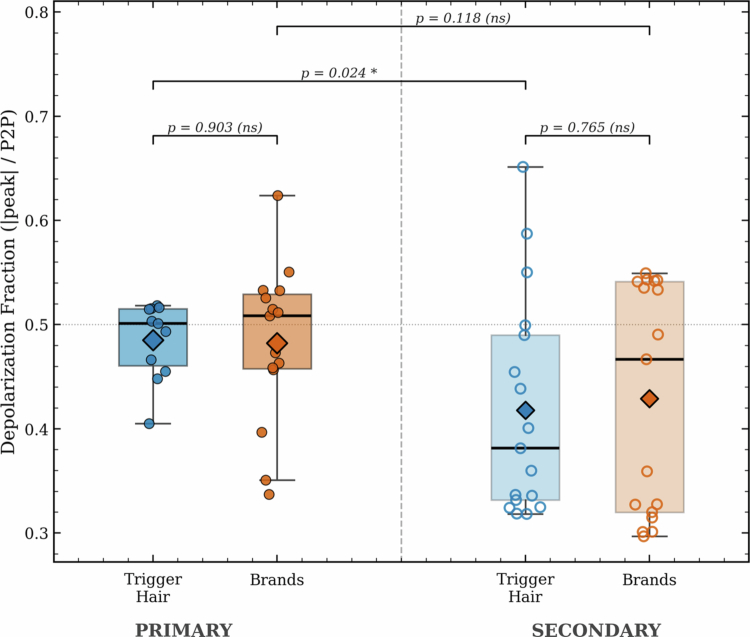
AP waveform shape by treatment and spike order (bandpass channel). Boxes show data tendency (median, IQR, whiskers to 10th/90th percentile) with individual data points showing Depolarization Fraction (|peak|/P2P) for Primary and Secondary spikes in each treatment group. Diamond symbols indicate group means. Pre-specified comparison: Primary TH vs. Primary Brands (*p =* 0.903, d = 0.04, ns). Exploratory comparisons: TH Primary vs. TH Secondary (*p =* 0.024, d = 0.79, Holm-corrected *p =* 0.168, ns); Secondary TH vs. Secondary Brands (*p =* 0.765, d = -0.10, ns); Brands Primary vs. Brands Secondary (*p =* 0.118, d = 0.56, ns). Dashed vertical line separates Primary and Secondary spike comparisons.

**Figure 5.**
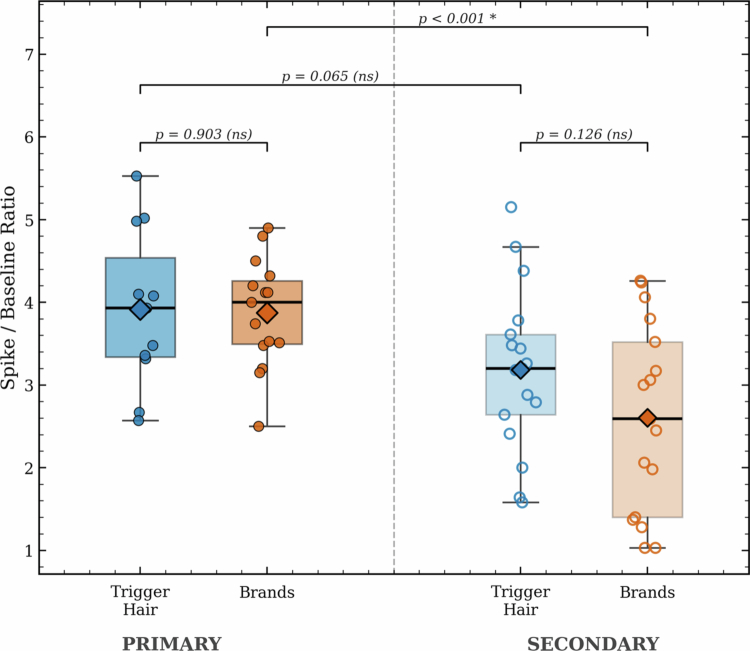
AP response amplitude by treatment and spike order (bandpass channel). Boxes show data tendency (median, IQR, whiskers to 10th/90th percentile) with individual data points showing Spike/Baseline Ratio for Primary and Secondary spikes in each treatment group. Diamond symbols indicate group means. Pre-specified comparisons: Primary TH vs. Primary Brands (*p* = 0.903, d = 0.05, ns); Brands Primary vs. Brands Secondary (*p* < 0.001, d = 1.34, Holm-corrected *p =* 0.0048, *). Exploratory comparisons: TH Primary vs. TH Secondary (*p =* 0.065, d = 0.75, ns); Secondary TH vs. Secondary Brands (*p =* 0.126, d = 0.54, ns). Dashed vertical line separates Primary and Secondary spike comparisons.

**Figure 6.**
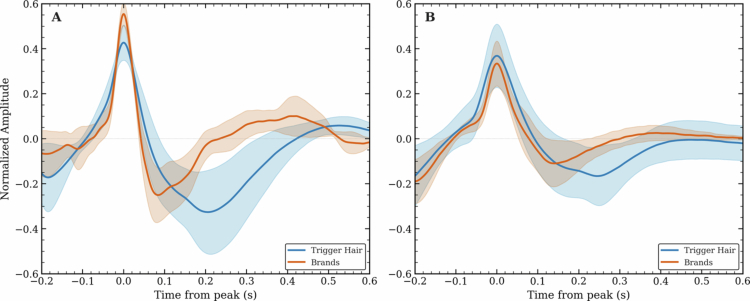
Mean action potential waveforms (± 95% CI) recorded from *Dionaea muscipula* trap lobes following mechanical trigger hair deflection (blue) and heated branding iron contact (orange). Waveforms were bandpass filtered (0.5–50 Hz), aligned on peak depolarization, and normalized to peak-to-peak amplitude to remove cross-recording calibration differences. The display window (−0.2 to 0.6 s relative to peak) captures the full asymmetric depolarization onset through repolarization recovery. (A) Primary APs (first spike per recording) (B) Secondary APs (subsequent spikes).

Primary Spike/Baseline Ratio did not differ significantly between TH and Brands stimulation in either the primary contact zone comparison (*p =* 0.903, d = 0.05; mean difference = + 0.042 A.U.; 95% CI [−0.668, + 0.752]; df = 16.6; Supplementary Data Sheet 3) or the pooled all-trials comparison (*p =* 0.337, d = 0.25; Figure 3). Primary Depolarization Fraction was similarly non-significant (*p =* 0.903, d = 0.04; mean difference = +0.003; 95% CI [−0.044, + 0.050]; df = 21.4; Figure 4). Primary and Secondary Spike/Baseline Ratios within the Brands treatment group differed significantly (*p* < 0.001, Holm-corrected *p =* 0.0048, d = 1.34; 95% CI on mean difference [+0.598, + 1.933]; Figure 5). Within-Brands Depolarization Fraction did not differ significantly between primary and secondary spikes (*p =* 0.118, d = 0.56; Figures 3 and 4). Within the TH group, Primary and Secondary Depolarization Fractions showed a nominally significant difference (*p =* 0.024, d = 0.79), whereas Primary and Secondary Spike/Baseline Ratios did not (*p =* 0.065, d = 0.75; Figures 4 and 5). Secondary spikes between-group comparisons did not reach significance for either Spike/Baseline Ratio (*p =* 0.126, d = 0.54) or Depolarization Fraction (*p =* 0.765, d = −0.10; Figures 4 and 5). Average waveform shapes were visually similar across treatments (Figure 6). Both stimulations produced nearly identical depolarization and repolarization peaks with broadly overlapping 95% confidence intervals. Secondary peaks showed reduced amplitude (waveform attenuation) and greater variability within treatment.

## Discussion

### Primary spike morphology

Primary spike morphology did not differ significantly between trigger hair stimulation and thermal petiole wounding. This null result at negligible effect size (d < = 0.05) strongly suggests that once any sufficient depolarizing perturbation reaches the trap, the trap generates its own stereotyped AP through its endogenous excitation machinery.^9^ Morphological equivalence between TH-evoked and wound-evoked primary spikes is therefore expected rather than remarkable. Both reflect the trap's canonical AP output, with waveform shape determined by the trap's own ion channel kinetics rather than by the arriving input's nature.^15^ The differences between primary and secondary spikes are consistent with ion channel fatigue during trap stimulation. The trap closure and detected AP are not a traveling electrical signal bypassing the trap-petiole electrical grounding, but a non-electrical signal initiating the trap's bioelectric mechanism.

A variation potential, driven by H+-ATPase inactivation and xylem pressure waves, can initiate trap closure.^5^ ^,^ ^20^ ^,^ ^21^ However, a passive signal traveling through the non-excitable petiole is unlikely to directly engage the trap's voltage-gated machinery to show canonical AP morphology at the electrode.^23^ Instead, thermal wounding likely disrupts petiole xylem tissue. This disruption creates a pressure surge through the fluid column shared by the petiole and the trap. The pressure surge presumably depolarizes trap mesophyll cells, which are the same cellular targets activated by trigger hair deflection.^12^ This depolarization then initiates trap-derived APs through the trap's own machinery.

Wound-evoked and TH-evoked primary spikes, then, resemble one another because they are the same signal type derived from different initiating mechanisms. The primary spike null result is evidence that the trap's closure threshold is accessible via sufficiently large, likely non-electrical, systemic perturbation. Future multi-electrode recordings spanning the petiole-trap junction would more directly characterize signal propagation and type.^23^ The significant within-Brands secondary spike attenuation (*p* < 0.001, Holm-corrected *p =* 0.0048) provides the more direct evidence against sustained canonical AP propagation from the petiole and is discussed below.

### Secondary signal propagation is consistent with non-regenerative propagation

The pre-specified within-Brands secondary spike Spike/Baseline Ratio attenuation (*p* < 0.001, Holm-corrected *p =* 0.0048, d = 1.34) provides the strongest evidence against canonical AP propagation from the petiole. Regenerative APs maintain constant amplitude during propagation.^4^ Graded signals attenuate with distance and time.^5^ The decaying perturbation brands pattern with preserved waveform shape is consistent with decaying subthreshold excitation across successive discharge events (Figure 6). Volkov^18^ characterized petiole-conducted signals as electrotonic rather than regenerative, consistent with this pattern. The secondary attenuation indicates that the wound-evoked perturbation weakens sufficiently after the primary discharge that subsequent threshold crossings become less reliable, consistent with a decaying non-regenerative input rather than a sustained AP reaction.

### Closure kinetics implicate hydraulic coupling

Among closing trials, wounded traps closed significantly slower than trigger hair-induced closure (*p =* 0.032, d = 1.01). Both treatments achieved substantial trap movement (> = 80% reduction in baseline opening). Brands closure trajectories split into two patterns. Brands-Fast trials resembled TH kinetics. Brands-Delayed trials achieved gradual closure over 2 to 7 seconds. This bimodal pattern may reflect variable perturbation strength. Stronger perturbations likely drove faster closure. Weaker perturbations produced the delayed pattern.

The VFT trap closes via a hydroelastic curvature mechanism.^25^ The upper leaf functions as a thin elastic shell maintained by differential hydrostatic pressure across two cell layers.^26^ External stimuli open pores between layers. Water redistributes from the upper to the lower layer and the bilayer snaps from convex to concave.^24–26^ Fluid transfer between layers, not pore opening, limits closure speed.^24^ ^,^ ^26^ HgCl_2_ inhibits trap closure after mechanical stimulation, confirming that aquaporin-mediated water transport is necessary for closure.^26^

Petiole thermal wounding likely disrupts hydraulic continuity between the petiole and the trap. Reduced continuity limits the water flux available to drive hydroelastic curvature. This explains the slower closure kinetics observed in the branding trials. However, hydraulic pressure across the petiole-trap junction was not directly measured in this study. Direct pressure measurements and aquaporin inhibition assays in wounded areas would test this interpretation more directly.

### A convergent threshold mechanism

Wounding stimulus introduces additional mechanistic considerations. Volkov et al.^27^ demonstrated that the electrical charge required to close the VFT trap decreases to approximately 30% of its room-temperature value at 28–36°C tissue temperature. At elevated temperatures (36–40°C) a single mechanical stimulus is sufficient for trap closure.^26^ ^,28^ Petiole thermal wounding elevates local and potentially systemic tissue temperatures. This lowers the trap's closure threshold concurrently with wound-evoked perturbation arrival. Trap closure following petiole wounding may therefore reflect convergent contributions from both the systemic perturbation signal and a thermally reduced closure threshold. This convergent mechanism also provides a plausible explanation for the slower Brands closure kinetics. A weaker wound-evoked signal combined with thermal threshold reduction may provide less energetic drive for rapid hydroelastic closure than a canonical trigger hair AP.

### The trap is isolated, but the system is integrated

These findings do not challenge the established electrical isolation framework.^9^ Secondary signal attenuation is inconsistent with regenerative AP propagation from the petiole. Inter-trap isolation under canonical wounding conditions remains well-supported.^19^ Electrical-hydraulic-chemical system perturbation can, however, engage the trap mechanism outside the canonical trigger hair pathway. These results extend rather than contradict electrical isolation.^9^ Trap isolation is real. The trap generates and propagates its own APs independently of the petiole.^18^ The trap's closure threshold is nonetheless accessible via non-AP systemic disturbances of sufficient magnitude.

Multiple trap impulses following single petiole wounding events warrant further investigation. The observed closure rate in this study is consistent with the AP-counting model of Böhm et al.^13^ This model dictates that two APs are required for trap closure and multiple impulses engage downstream JA signaling cascades and activate digestive enzyme secretion. Our data suggests that thermal petiole wounding similarly but less reliably engages the counting mechanism to initiate trap closure. Whether thermal wounding of the petiole can engage the full AP-counting cascade remains an open question. If so, a non-prey stimulus could activate prey-processing responses at measurable physiological cost.^17^ Thermal stressors such as fire represent a plausible ecological pressure for coevolution of trap sensitivity in fire-maintained habitats.

Huang & Hedrich^22^ recently identified Ca^2+^ waves originating from the trigger hairs as the driver of thermal sensitivity in *D. muscipula.* Our results underline heat sensitivity as a physiological influence in the species and may further support ecological connections between light-dependent carnivory and wildfire-dependent overstory clearing of the species’ native range.^29^ ^,^ ^30^ Frequent fires are necessary for *D. muscipula* natural persistence and influence trap growth.^31^ However, the relevance of the branding temperatures in comparison to natural fire conditions is limited. Past heat experiments relied on relatively low temperatures (~50 °C) and burning matches to simulate swamp fires and heat stress.^22^ Several studies indicate that trigger hairs and associated chemical gradients are clearly sensitive to even ~38 °C.^22^ ^,^ ^32^ ^,^ ^33^ The soldering-iron-driven temperature stress examined here is clearly both more extreme and more focused on a smaller area than previous experiments and natural conditions. In future experiments, other researchers could use a range of lower temperatures and large surface area instruments to examine temperature stress on the petiole and other areas. Fire is clearly both physiologically and ecologically relevant but careful consideration must be made in how thermal stress is applied to ensure that conditions match natural conditions. Perhaps a larger, lower temperature, hot instrument could be applied to the petiole to mimic burning grasses and interrogate if thermal petiole injury can close traps in natural settings.

The hydraulic mechanism proposed here also connects to broader questions about the evolutionary origin of carnivory. Carnivory in plants is thought to have evolved from ancestral anti-herbivory mechanisms.^34^ ^,^ ^35^ A piercing or sucking arthropod feeding on VFT petiole tissue could interrupt xylem continuity, potentially generating a hydraulic pressure transient capable of initiating the electrical cascade described here. The trap's sensitivity to xylem disruption may reflect an anti-herbivory response co-opted for prey capture.

## Conclusion

Thermal petiole wounding initiates count-specific systemic electrical perturbations sufficient to trigger trap closure. Closure is unexpected given the established electrical isolation of the VFT trap. The bimodal Brands closure pattern may reflect variation in wound severity, wound location precision, or individual plant state dictating the number of electrical signals fired. Multiple sequential electrical impulses occurred in a subset of branding trials, consistent with the AP-counting model of Böhm et al.,^13^ in which two APs suffice for closure and additional APs engage downstream JA signaling cascades.

Primary spike morphology did not differ significantly between trigger hair-evoked and wound-evoked events. We interpret this as consistent with the trap generating its own stereotyped AP once any sufficient perturbation arrives, not as evidence that the two signals are identical. Confidence intervals for both primary comparisons are wide at the present sample sizes (*n =* 11 TH, *n =* 15 Brands; Supplementary Table S3). Formal equivalence cannot be claimed. Within-Brands primary-to-secondary attenuation, the sole comparison surviving multiple comparisons correction, confirms that wound-evoked signals degrade across sequential trap events. The integrated electrical-hydraulic-chemical system can engage the isolated trap mechanism.

These results suggest that the VFT's electrically isolated trap is more accessible to systemic disruption than the isolation framework alone implies. However, the underlying mechanism that drove the observations made in this study is currently unknown. Other researchers should focus on conducting similar experiments that directly measure the hydraulic changes, xylem disruption, pressure, and other responses implicated here. Thermal artifact from the heated instrument cannot be fully excluded and should be addressed in future work with non-contact thermal stimulation controls. Changing trap sensitivity and indirect electrical responses in the trap cannot be excluded. Moreover, although lobe separation pixel distance is a useful proxy for closure extent, it is subject to computer vision limitations. Trap geometry is not perfectly linear and trigonometric corrections were not applied. These closure metrics should be interpreted as comparative rather than absolute kinematic values.

The integrated nature of the *D. muscipula* electrical system deserves further exploration. This study represents the first step in our ongoing investigation of plant bioelectricity. With citizen-science-like investigations such as those documented in Madariaga et al.^10^ and decreasing costs associated with physiological measurements, plant bioelectric systems are more accessible than ever before. Future investigations should employ multiple probes to examine if systematic perturbations can elicit adjacent traps within the leaf rosette and if injured petiole bioelectric/hydraulic systems can heal from thermal wounds over time. *D. muscipula* physiology is an opportunity for multi-disciplinary investigations and future studies could integrate computer vision, biochemistry, electrophysiology, and more.

## Supplementary Material

Supplementary Material Supplementary Data.

VFT_Supplementary_Data.xlsx VFT_Supplementary_Data.xlsx

## Data Availability

Supplementary datasets associated with this manuscript are publicly available online through Zenodo. Electrophysiology measurements used to produce the figures in this manuscript are available at doi: 10.5281/zenodo.20466875. Ten representative electrophysiology recordings including five derived from trigger hair deflections and five from branding stress are available at doi: 10.5281/zenodo.21048106.
