# Supplementary material for: Thermal petiole wounding triggers trap closure in Dionaea muscipula
Source: Plant Signal Behav. 2026 Jul 9;21(1):2700900. doi: 10.1080/15592324.2026.2700900 (PMC13353776; doi:10.1080/15592324.2026.2700900)
Supplement: Supplementary Material — Supplementary Data. [file KPSB_A_2700900_SM8287.docx]

**Supplementary Data**

All data supporting the figures and statistical analyses are provided in a single supplementary workbook (VFT_Supplementary_Data.xlsx) with four sheets.

**Sheet 1.** Spike Details: Individual spike-level records for all TriggerHair and Brands detections. Columns: Treatment, Filename, Spike_Num, Spike_Order (Primary/Secondary), BP_Peak, BP_Trough, BP_P2P (bandpass-filtered channel, arbitrary units), Spike_RMS, Spike_Baseline_Ratio, Depol_Fraction, Analysis_Status (included/excluded). *n =* 63 raw detections across 26 contributing recordings (11 TH, 15 Brands); *n =* 60 working spikes after exclusion of 3 outliers (1 spike/baseline ratio > 7.0; 2 depolarization fraction > 0.95; see Methods). Depolarization Fraction is computed as |BP_Peak| / BP_P2P.

**Sheet 2.** File Summary: Per-recording summary statistics. Columns: Treatment, Filename, N_Spikes_Raw, N_Spikes_Working, Primary_Ratio, Primary_Depol, Mean_Secondary_Ratio, Mean_Secondary_Depol. Provides recording-level overview for all 28 TriggerHair and Brands recordings in the pipeline output.

**Sheet 3.** Statistical Tests: Complete Welch's t-test results for all eight pairwise comparisons reported in the manuscript. Columns: Metric, Comparison, Designation (Pre-specified/Exploratory), n1, Mean1, SD1, n2, Mean2, SD2, Welch's_t, p_value, Cohen's_d, CI95_lower, CI95_upper, Holm_p, Holm_sig. Holm–Bonferroni correction applied across all eight comparisons. Key results: Within-Brands Primary vs. Secondary SBR p < 0.001 -> Holm *p =* 0.0048 (pre-specified, significant); 95% CI [+0.598, +1.933]. Primary TH vs. Brands SBR *p =* 0.903 -> Holm *p =* 1.000 (pre-specified, ns); 95% CI [-0.668, +0.752]. Primary TH vs. Brands DepFrac *p =* 0.903 -> Holm *p =* 1.000 (pre-specified, ns); 95% CI [-0.044, +0.050].

**Sheet 4.** Closure Trials: Per-trial closure summary. Columns: Trial, Treatment, Closed (Yes/No), T50_s (time to 50% closure in seconds), Group (Touch / Brands-Fast / Brands-Delayed / No Closure), n_frames. T50 threshold for Fast/Delayed classification: 1.5 s (see Methods). *n =* 19 Brands trials (12 closed: 6 Fast, 6 Delayed; 7 no closure); *n =* 6 Touch trials (5 closed, 1 no closure).
